# Near-Infrared Fluorescent pH Responsive Probe for Targeted Photodynamic Cancer Therapy

**DOI:** 10.1038/s41598-020-58239-5

**Published:** 2020-01-28

**Authors:** Siriwalee Siriwibool, Nantawat Kaekratoke, Kantapat Chansaenpak, Kittipan Siwawannapong, Pannipa Panajapo, Kritsana Sagarik, Parinya Noisa, Rung-Yi Lai, Anyanee Kamkaew

**Affiliations:** 1 School of Chemistry, Institute of Science, Suranaree University of Technology, Nakhon, Ratchasima 30000 Thailand; 2 National Nanotechnology Center, National Science and Technology Development Agency, Thailand Science Park, Pathum Thani, 12120 Thailand; 3 Laboratory of Cell-Based Assays and Innovations, School of Biotechnology, Institute of Agricultural Technology, Suranaree University of Technology, Nakhon Ratchasima, 30000 Thailand; 4 Center of Excellence in Advanced Functional Materials, Suranaree University of Technology, Nakhon Ratchasima, 30000 Thailand

**Keywords:** Targeted therapies, Organic chemistry, Single-molecule fluorescence

## Abstract

We developed a pH dependent amino heptamethine cyanine based theranostic probe (**I**_**2**_**-IR783-Mpip**) that can be activated by near infrared light. **I**_**2**_**-IR783-Mpip**, in acidic condition, exhibited an intense, broad NIR absorption band (820–950 nm) with high singlet oxygen generation upon exposure to NIR light (~850 nm). Theoretical calculations showed that the protonation of the probe in an acidic environment decreased the molecular orbital energy gaps and increased the intramolecular charge transfer efficiency. **I**_**2**_**-IR783-Mpip** exhibited good photodynamic efficiency towards liver hepatocellular carcinoma cells under physiological and slightly acidic conditions while normal human embryonic kidney cells remained alive under the same conditions. Detection of intracellular reactive oxygen species (ROS) in cells treated with **I**_**2**_**-IR783-Mpip** after NIR light exposure confirmed PDT efficiency of the probe in acidic environment. Moreover, **I**_**2**_**-IR783-Mpip** also demonstrated efficient phototoxicity under deep-seated tumour cell system. We believed this is the first PDT agent that possesses intrinsic tumour binding and selectively eradicate tumour in acidic environment under 850 nm NIR lamp.

## Introduction

Photodynamic therapy (PDT) is an attractive non-invasive technique for treating cancers. Extensively, two steps are involved in PDT including, (i) delivery of a photodynamic agent to tumours and (ii) irradiation of tumour sites with specific light to activate PDT agent followed by reactive oxygen species (ROS) generation that triggers cancer cell death^1^. In general, cancer cells behave differently by comparison with normal cells. For example, the lack of oxygen in tumour creates strong hypoxia condition leading to lactic acid build-up and lower extracellular pH level in tumour environment (pH 6.2–6.9)^2,3^. In addition, the lysosomal pH in cancer cells (pH_lys_ 3.8–4.7) shows higher acidity than that in normal cells (pH_lys_ 4.5–6.0)^4^.

Near-infrared fluorescent dyes that can absorb and emit light in a range 700–1000 nm are good for tumour detection at millimetre depth due to less background fluorescence from endogenous molecules^5,6^. Among them, heptamethine cyanines (Hcyanines) were used extensively as tools for cancer imaging because of their high fluorescence quantum yields, great photothermal and/or photodynamic effect and good biocompatibility^7,8^. Moreover, Hcyanines could be internalized by cancer cell through organic anion-transporting polypeptides (OATPs), a group of cell membrane-bound solute carriers^9–11^. OATPs could mediate the cellular transport of drug and exogenous materials^12^. The uptake of Hcyanines in cancer cells was concerted actions exerted by hypoxia and activation of HIF1alpha/OATPs signalling leading to enhance dye uptake, but little to no accumulation in normal cells due to low expression of OATPs^13^.

Recently, some nanomaterials based Hcyanines were reported to target acidic tumour environment with therapeutic effects^14,15^. However, only few Hcyanines have been reported as the stand-alone small molecules targeted cancer that were pH sensitive and generated heat after activation (so-called photothermal effect) or produced PDT effect. For example, IR2 was reported as a pH switchable NIR fluorescence and photothermal agent by adjusting the intramolecular charge transfer (ICT) efficiency using dimethylamine group. By this way, IR2 could selectively visualize and eradicate cancer cells^16^. In another study, Hcyanine was conjugated with rhodamine via disulphide linkage^17^. The probe, RhoSSCy, possessed dual-responsive to both thiols and pH with NIRF/PA dual-modal imaging, and NIR-sensitizing PDT activities *in vitro* and *in vivo*. Therefore, development of theranostic probes for precisely locating and eliminating tumour are still valuable for clinical translations.

In this work, we developed a Hcyanine based theranostic probe (**I**_**2**_**-IR783-Mpip**) for NIR fluorescent imaging and PDT that aimed to target cancer cells via OATPs and the protonation in acidic tumour environment. Our probe showed remarkably red-shifted NIR absorbance in acidic pH, which has not been reported elsewhere. These phenomena are beneficial for activating such PDT agent using long wavelength of light, i.e. 850 nm, to reach highest possible penetration depth in NIR-I region^6^ while high fluorescence is still remained.

## Results

### Synthetic procedures

**Scheme 1:**
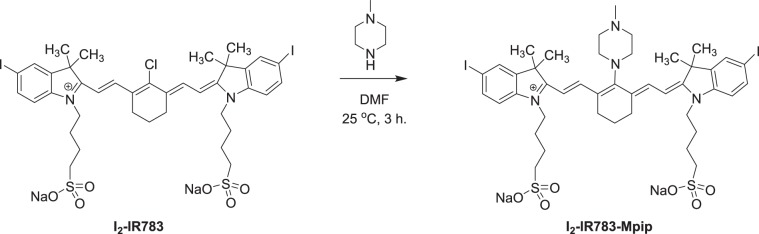
Synthesis of **I**_**2**_**-IR783-Mpip**.

**I**_**2**_**-IR783-Mpip** was synthesized via conjugate addition reaction between I_2_-IR783^18^ and *N*-methylpiperazine (Scheme 1). Hcyanine derivative IR783 was selected as a NIR fluorophore because of its good biocompatibility, optimized emission wavelength and intracellular uptake via OATPs. In addition, the presence of iodine atoms onto the 5-position of both terminal indole rings leads to enhancing PDT effect^18^. Substitution of *N*-methylpiperazine group on the meso position of I_2_-IR783 results in achieving a pH response. At neutral pH, the fluorescence of **I**_**2**_**-IR783-Mpip** is expected to be quenched by the effect from the nitrogen lone pair electrons of *N*-methylpiperazine moiety through a photoinduced electron transfer (PeT) process^19^. While in acidic environments, protonation of the nitrogen atoms will block the PeT process causing the increased fluorescence signal. This compound was fully characterized by ^1^H and ^13^C NMR spectroscopy and high-resolution mass spectrometry (HRMS) (Fig. S1 in ESI†).

### Photophysical properties

**Figure 1:**
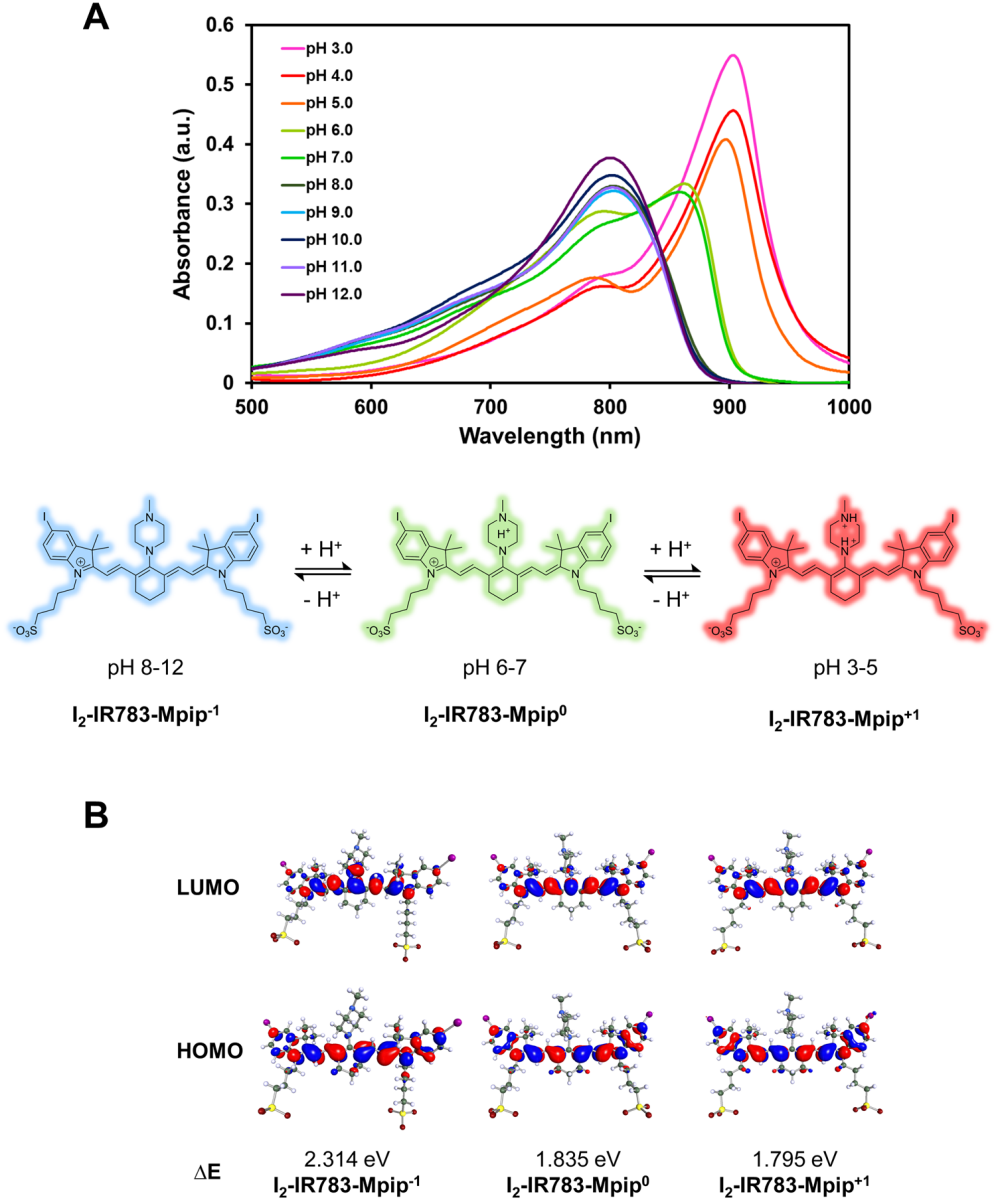
(**A**) Vis**-**NIR spectra of **I**_**2**_**-IR783-Mpip** and proposed structures of **I**_**2**_**-IR783-Mpip** in different pH **(**3.0**–**12.0**)**. (**B**) Equilibrium structures of I_2_-IR783-Mpip^-1^, I_2_-IR783-Mpip^0^ and I_2_-IR783-Mpip^+1^ obtained from DFT/6-311 G geometry optimizations in the COSMO phase, dielectric environment 78. The values of the HOMO and LUMO isosurfaces are 0.025.

Optical properties of the dye were found to be subjectable to the pH of the solution. **I**_**2**_**-IR783-Mpip** absorbed light from visible to NIR region (500–920 nm), peaking at 860 nm under pH 6–7. When two nitrogen atoms of *N*-methylpiperazine moiety on **I**_**2**_**-IR783-Mpip** were protonated under acidic environments (pH 3.0–5.0), absorption spectra were red shifted (Fig. 1A). On the other hand, once **I**_**2**_**-IR783-Mpip** exposed to basic environments (pH 8.0–12.0), absorption spectra were blue shifted. These phenomena could be the results from an intramolecular charge transfer (ICT) in **I**_**2**_**-IR783-Mpip**. Since the **I**_**2**_**-IR783-Mpip** contains both electron donor (amine) and acceptor (Hcyanine), a charge separation is obtained within the fluorophore. The electron-donating ability of the donor at the meso-position in a cyanine scaffold would cause absorption and/or fluorescence spectra shift^20^. In addition, based on the reported pKa values of sulfonic acid (pKa = −2.6) and *N*-methylpiperazine (pKa = 3.81 and 8.38)^21^, the molecular structures were proposed in three forms, I_2_-IR783-Mpip^−1^, I_2_-IR783-Mpip^0^, and I_2_-IR783-Mpip^+1^ in solutions pH 8–12, 6–7 and 3–5, respectively (Fig. 1A).

To obtain fundamental information on the effect of pH and charge states of **I**_**2**_**-IR783-Mpip** on the absorption spectra in Fig. 1A, quantum chemical calculations based on the density functional theory (DFT) method was applied using the 6–311 G basis set in the COSMO phase, dielectric environment 78. Equilibrium structures of the deprotonated and protonated forms of **I**_**2**_**-IR783-Mpip**^**n**^ (n = −1, 0 and +1) in the electronic ground state were computed using TURBOMOLE software package^22^ as shown in Fig. 1B. Frontier molecular orbital from DFT/6-311 G showed the HOMO and LUMO molecular orbitals of I_2_-IR783-Mpip^−1^, I_2_-IR783-Mpip^0^, and I_2_-IR783-Mpip^+1^, possible species that existed in solutions pH ranging from 3–12. The changes of the HOMO and LUMO in these three structures revealed that electron density on iodinated-indole rings was distributed to the IR783 backbone after excitation. In addition, the localized electron on iodinated-indole ring on the HOMO was gradually more dominant by the increase of total structural charge, suggesting the existence of a stronger charge transfer to IR783 core in acidic conditions. Therefore, the greater electron distribution from I_2_-IR783-Mpip^−1^ to I_2_-IR783-Mpip^+1^ lead to decrease energy gap of 0.519 eV, causing the red-shift in the absorption spectra in acidic solutions. Furthermore, as confirmed by a reduced energy gap of 0.479 eV, structure with balanced charge, I_2_-IR783-Mpip^0^, was proposed as an existing form in solution pH 6–7 where the absorbance spectra were shown in the middle (Fig. 1A). In addition, the calculated energies of I_2_-IR783-Mpip^0^ with different protonated sites of *N*-substituted piperazine suggested that proton could possibly add to either nitrogen atom in the piperazine ring since the total energies of the two protonated forms are not significantly different (Fig. S2).

**Figure 2:**
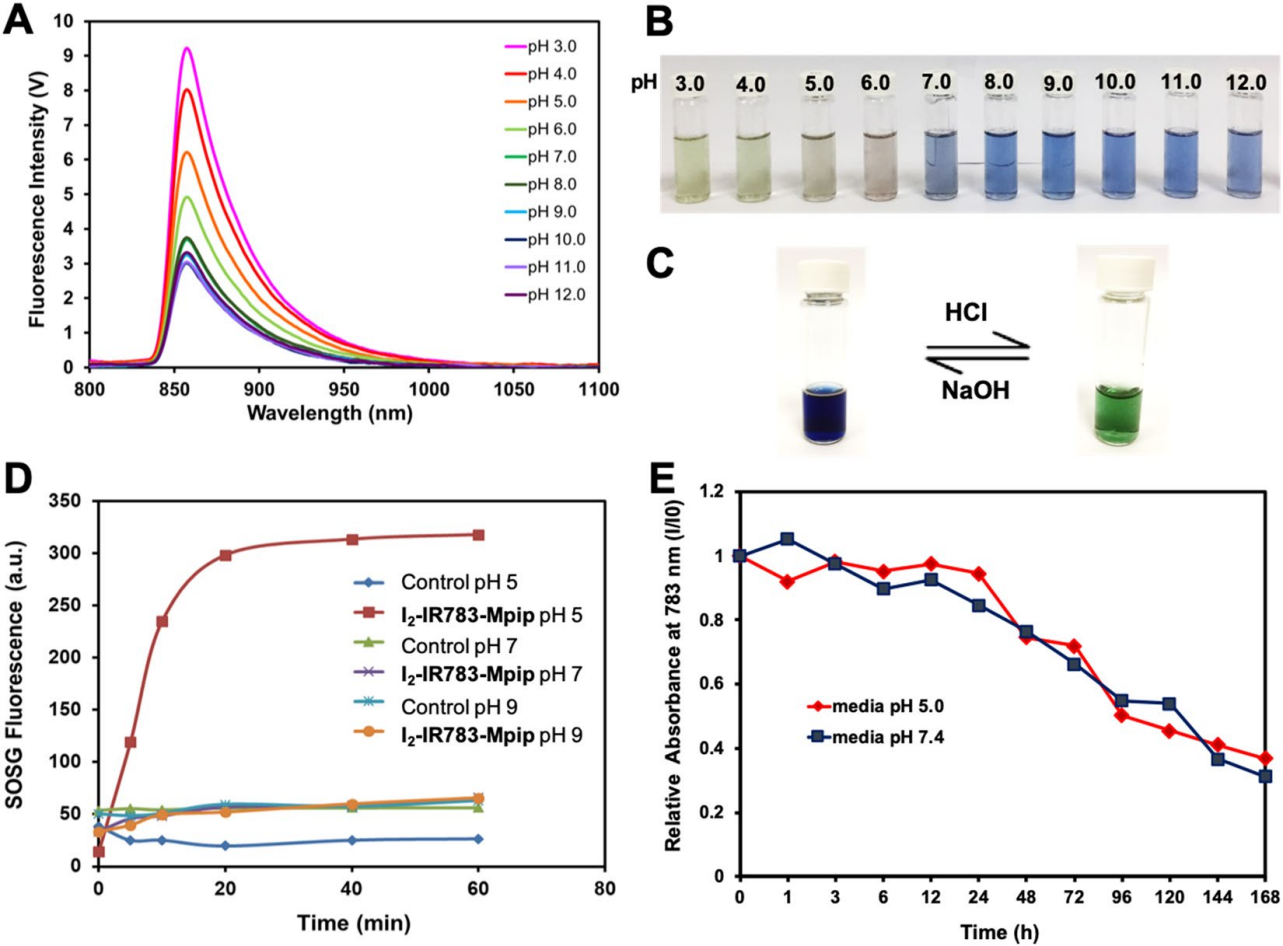
Optical properties of **I**_**2**_**-IR783-Mpip**. (**A**) Fluorescent spectra of **I**_**2**_**-IR783-Mpip** in different pH (3.0–12.0) under excitation at λ 850 nm. (**B**) Solutions of **I**_**2**_**-IR783-Mpip** in different pH (3.0–12.0). (**C**) Reversible colour changes of **I**_**2**_**-IR783-Mpip** in methanol solution. (**D**) Singlet oxygen generation of **I**_**2**_**-IR783-Mpip** upon irradiation with 850 nm lamp up to 60 min. (**E**) Stability tests of **I**_**2**_**-IR783-Mpip** in cell culture media pH 5.0 (red line) and pH 7.4 (blue line) at 37 °C for up to 7 days.

Next, to target tumour environments, **I**_**2**_**-IR783-Mpip** was excited at 850 nm, where the lowest background signal can be obtained^23,24^, to activate the molecules in neutral and acidic conditions. As expected, strong emission intensities were only observed from the solution in pH < 7.0 (Fig. 2A). These results implied that **I**_**2**_**-IR783-Mpip** could be selectively detected at different pH. In basic environments, the solutions turned to blue colour whereas in acidic environments (pH < 6.0), the solutions have gradually changed to green colour which varies according to the light absorptions in the Vis-NIR absorbance spectra (Fig. 2B). Moreover, our probe demonstrated reversible optical responses in acidic and basic environments. As shown in Fig. 2C, when aqueous solution of HCl was dropped into an alcoholic solution of **I**_**2**_**-IR783-Mpip**, protonation occurred at the two nitrogen atoms of *N*-methylpiperazine resulting in reducing of the electron-donating ability of the two nitrogen atoms causing a red shift of the absorption spectra to around 900 nm which made the solution turned green. On the other hand, a few drops of aqueous NaOH caused the protonated solution reversed back to the blue colour where the deprotonated form appeared. These results suggested that **I**_**2**_**-IR783-Mpip** could act as a reversible colour pH probe.

As the red shift of the spectra occurred after protonation, ^1^O_2_ generation of **I**_**2**_**-IR783-Mpip** upon activation by NIR LED lamp under various conditions was also investigated. Singlet oxygen sensor green (SOSG) was used to detect ^1^O_2_ that produced from the light-triggered reaction. After irradiating **I**_**2**_**-IR783-Mpip** with NIR light (850 nm, light intensity 30 mW/cm^2^) for 60 min in buffer solutions pH 5.0, pH 7.0 and pH 9.0, emission intensities of SOSG (at 510 nm) increased only in the case of the acidic solution containing **I**_**2**_**-IR783-Mpip** (Fig. 2D). The results suggested that ^1^O_2_ was generated over irradiated time when **I**_**2**_**-IR783-Mpip** was in the acidic condition (pH 5.0) whereas significantly less amount of ^1^O_2_ was generated in neutral and basic conditions. Moreover, singlet oxygen quantum yield (Φ_Δ_) of **I**_**2**_**-IR783-Mpip** under 850-nm excitation was found to be 0.12 and 0.10 in buffer pH 5.0 and 7.0, respectively, compared to methylene blue (Fig. S3). Therefore, it is possible to apply our pH probe for cancer targeting since the tumour environments are usually acidic. Moreover, this probe could be toxic to tumour cells by generating ^1^O_2_ only when it exposes to NIR light, 850 nm.

Before moving to *in vitro* experiments, stability of **I**_**2**_**-IR783-Mpip** was also investigated in cell culture media pH 5.0 and pH 7.4 at 37 °C for up to 7 days (Fig. 2E). It was found that, **I**_**2**_**-IR783-Mpip** was stable under these conditions for 24 h and the NIR absorptions gradually decreased from day 2 to day 7. Notably, **I**_**2**_**-IR783-Mpip** exhibited similar stability profile in both cell culture media. Moreover, cell viability assay of HepG2, a human liver cancer cell line, was performed to confirm the effect of pH in the cell culture media. It was found that HepG2 cells maintained full viability after exposure to media pH 5.0 for up to 12 h. After 24 to 36 h incubation with media pH 5.0, the cells remained about 70 % viability (Fig. S4). This implied that long time exposure to pH 5.0 media affected cell viability.

### Cell internalization

**Figure 3:**
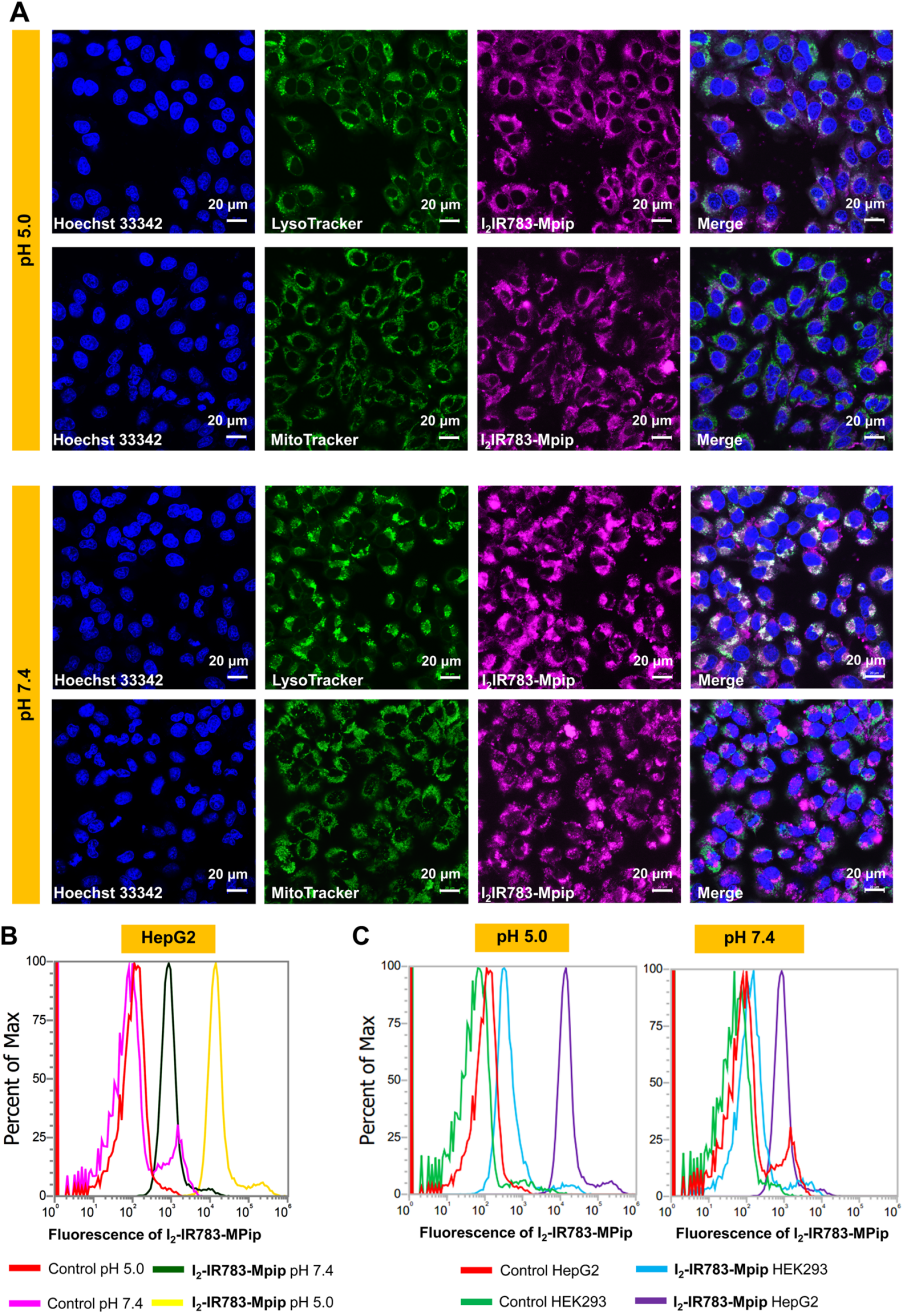
(**A**) Confocal images of HepG2 cells incubated with 30 μM of **I**_**2**_**-IR783-Mpip** for 6 h and colocalization of **I**_**2**_**-IR783-Mpip** with LysoTracker green (Pearson’s R value = 0.68 in pH 5.0 and 0.68 in pH 7.4) and MitoTracker Green (Pearson’s R value = 0.31 in pH 5.0 and 0.45 in pH 7.4). (**B**) Flow cytometry of HepG2 cells incubated with 15 μM of **I**_**2**_**-IR783-Mpip** for 12 h in culture media pH 5.0 and 7.4. (**C**) Flow cytometry of HepG2 and HEK293 cells incubated with 15 μM of **I**_**2**_**-IR783-Mpip** for 12 h in culture media pH 5.0 and 7.4. Scale bars = 20 μm.

*In vitro* experiments were performed to confirm the application of **I**_**2**_**-IR783-Mpip** in cancer cell treatment. Cell internalization and colocalization experiments were undertaken to visualize favored organelles for accumulation of **I**_**2**_**-IR783-Mpip** in HepG2 cells. As displayed in confocal images, **I**_**2**_**-IR783-Mpip** was internalized inside HepG2 and colocalized to some degree with MitoTracker Green (Pearson’s R value = 0.31 in pH 5.0 and 0.45 in pH 7.4) and much more with LysoTracker green (Pearson’s R value = 0.68 in pH 5.0 and 0.68 in pH 7.4, Figs. 3A and S5). These indicated that the probe preferred to accumulate mostly in lysosomes and less in mitochondria, which is beneficial for the photosensitizer that can be activated in the acidic pH since in cancer cells, the lysosomal pH_lys_ is about 3.8–4.74. Under physiological pH, **I**_**2**_**-IR783-Mpip** could be transported by OATP membrane proteins^12^. However, in the acidic environment, pH 3–5, the chemical structure of **I**_**2**_**-IR783-Mpip** is presented in a form of positive charges (+1), therefore, the preferable uptake mechanism of the dye at pH 5.0 could be via an active transport manner, including ATP-driven transport, but not via the OATPs. Moreover, there have been reported that the uptake mechanism of some cyanine derivatives, i.e. IR783, are related to micropinocytosis^13,25^.

In addition, time dependent internalization of **I**_**2**_**-IR783-Mpip** in cancer cells (HepG2) was performed in both culture media pH 5.0 and 7.4. Confocal images revealed that the uptake of **I**_**2**_**-IR783-Mpip** in HepG2 cells obviously increased within the first 12 h of incubation in both culture media (Fig. S6). Subsequent, to investigate tumour selectivity of **I**_**2**_**-IR783-Mpip**, quantitative cell internalization analysis of the probe in cancer cells and normal cells was performed using flow cytometry. At different pH, the uptake of **I**_**2**_**-IR783-Mpip** by HepG2 cells was much higher in media pH 5.0 than that in media 7.4 (Fig. 3B). By comparison, at concentration of 15 μM and 12 h incubation, the uptake of **I**_**2**_**-IR783-Mpip** in normal cells (Human embryonic kidney 293 cells, HEK293) was significantly less than the one in HepG2 cells in both media conditions (pH 5.0 and pH 7.4, Fig. 3C). Therefore, **I**_**2**_**-IR783-Mpip** could be a cancer targeted agent that have potential in selective destroying cancer cells via PDT.

### Photodynamic therapy

**Figure 4:**
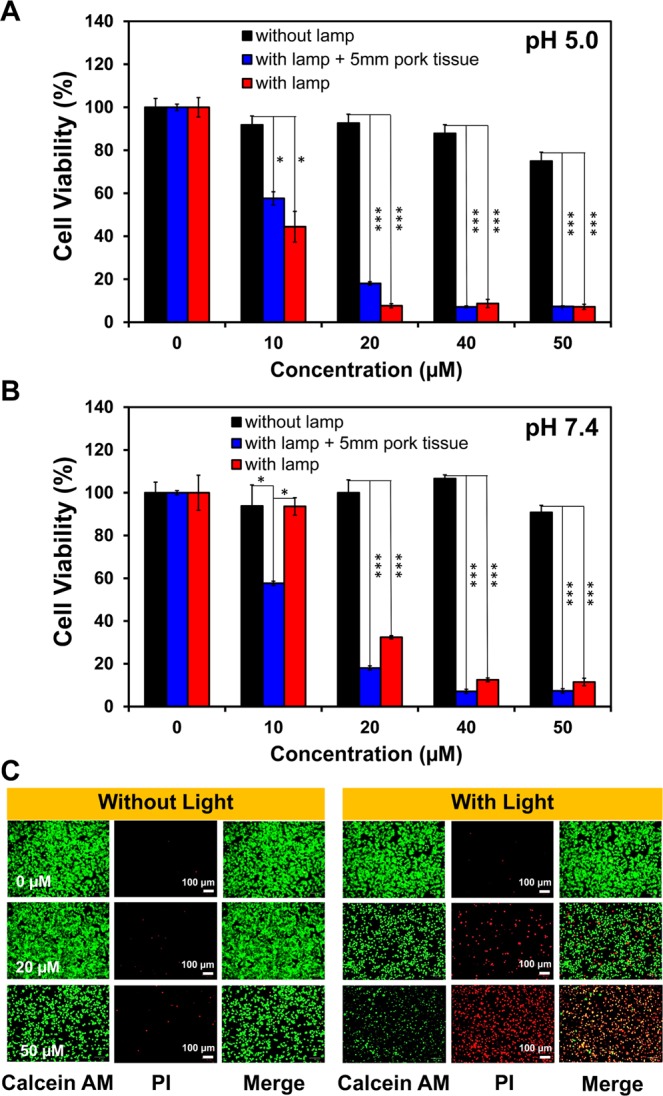
Photodynamic therapy effect of **I**_**2**_**-IR783-Mpip**. Relative viabilities of HepG2 cells in media (**A**) pH 5.0 and (**B**) pH 7.4; red bars represent cells irradiated with 850 lamp, blue bars represent cells covered by 5 mm pork tissue with 850 nm light irradiation and black bars represent cells without irradiation. (**C**) LIVE/DEAD viability/cytotoxicity assay of **I**_**2**_**-IR783-Mpip** under dark and light conditions at various concentrations (0, 20 and 50 µM). Scale bars = 100 μm. Statistical analysis are based on one-way ANOVA followed by Tukey’s post-hoc analysis (*P < 0.05, **P < 0.01, ***P < 0.001).

In general, PDT agents should be harmless in the dark state but highly toxic once being exposed to the specific light. To study the photocytotoxicity of **I**_**2**_**-IR783-Mpip**, HepG2 cells were incubated with various concentrations of **I**_**2**_**-IR783-Mpip** for 6 h in both media pH 5.0 and pH 7.4, exposed to 850 nm LED light (30 mW/cm^2^) for 30 min, and then re-incubated in the dark for another 12 h, respectively. Relative viabilities of the cells exposed to 850 nm light were confirmed to be inversely proportional to the concentrations of **I**_**2**_**-IR783-Mpip** in both culture media conditions. Cell viability assay revealed that no significant dark cytotoxicity of **I**_**2**_**-IR783-Mpip** was observed even at high concentration up to 50 µM in both culture media (pH 5.0 and pH 7.4, Fig. 4A,B). In contrast, normal cells HEK293 incubated with **I**_**2**_**-IR783-Mpip** maintained viability even after 850 nm light exposure for 30 min in both culture media (Fig. S7), indicating that **I**_**2**_**-IR783-Mpip** was selectively toxic to cancer cells after illumination.

To investigate whether the NIR absorbed **I**_**2**_**-IR783-Mpip** could be activated in deep-seated tumour cells, a piece of 5 mm pork tissue was placed between cells and the light source. After light exposure, HepG2 cell viabilities decreased in a dose dependent manner (Fig. 4A,B), which were similar to those results when there was no pork tissue covered on the cells. These observations strongly suggested that **I**_**2**_**-IR783-Mpip** could selectively destroy deep-seated cancer cells when it was irradiated with 850 nm LED light for 30 min in both physiological and slightly acidic environments.

To further confirm the effectiveness of **I**_**2**_**-IR783-Mpip** in terminating cancer cells under light irradiation, LIVE/DEAD viability/cytotoxicity assay was performed. HepG2 cells were plated in a 6-well plate and incubated for 24 h before adding **I**_**2**_**-IR783-Mpip**, at 20 µM and 50 µM, and then re-incubated for another 6 h. After that, the cells were exposed to 850 nm light for 30 min before staining with calcein AM and propidium iodide (PI). Calcein AM can easily enter the live cells and emit a strong green fluorescence after being excited with a 495 nm laser, whereas PI can easily enter the dead cells, bind to DNA by intercalating between the bases and emit a strong red fluorescence after being excited with a 535 nm laser. Visualization by an inverted fluorescence microscope, the increased red fluorescence was observed with the increased amount of **I**_**2**_**-IR783-Mpip** incubated in cells after irradiation with 850 nm light, indicating that more dead cell populations occurred when increasing the dose of **I**_**2**_**-IR783-Mpip**. Fluorescence from Calcein AM and PI co-stained cells confirmed the effectiveness of photodynamic cancer cell ablation induced by **I**_**2**_**-IR783-Mpip** in media at pH 5.0 (Fig. 4C) and pH 7.4 (Fig. S8).

**Figure 5:**
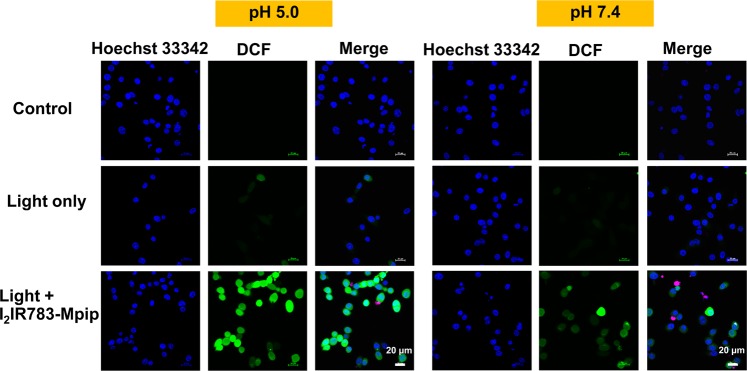
Detection of intracellular reactive oxygen species generated by **I**_**2**_**-IR783-Mpip** (20 μM) at pH 5.0 and 7.4 in HepG2 cells using DCFDA cellular ROS detection assay. Scale bars = 20 μm.

Furthermore, intracellular reactive oxygen species (ROS) generation was also observed from the cell culture contained **I**_**2**_**-IR783-Mpip** after irradiated with 850 nm light. HepG2 cells were treated with **I**_**2**_**-IR783-Mpip** for 6 h in both media (pH 5.0 and 7.4), stained with 2,7-dichloro-dihydro-fluorescein diacetate (DCFH-DA) and then exposed to 850 nm light for 20 min. DCFH-DA can be oxidized by intracellular ROS to produce 2,7-dichloro-dihydro-fluorescein (DCF). Intracellular DCF can be detected by fluorescence confocal microscopy using 488 nm excitation to obtain a strong green fluorescence. The confocal images showed the production of DCF was significantly increased in the presence of light exposure and **I**_**2**_**-IR783-Mpip** in both culture media but more obvious in media pH 5.0 than the one in pH 7.4 (Fig. 5). In addition, higher amount of green fluorescence could be detected from more acidic environment (Fig. S9).

## Conclusions

In summary, **I**_**2**_**-IR783-Mpip** was successfully developed as a pH sensitive theranostic agent for fluorescent imaging and PDT. The probe exhibited red-shifted Vis-NIR absorbance spectra under acidic conditions and blue-shifted in basic conditions. Upon exposure to 850 nm LED light for 30 min, **I**_**2**_**-IR783-Mpip** generated higher amount of ^1^O_2_ in more acidic environment. Moreover, **I**_**2**_**-IR783-Mpip** selectively internalized cancer cells and the uptake was in dose- and time-dependence manners. At concentration as low as 20 µM, **I**_**2**_**-IR783-Mpip** showed light induced cytotoxicity to HepG2 cells resulting to about 10 and 30 % viabilities in acidic and physiological conditions, respectively. In addition, under simulated deep-tissue setting, **I**_**2**_**-IR783-Mpip** still maintained its PDT efficiency towards cancer cells. Better reactive oxygen species generation of **I**_**2**_**-IR783-Mpip** was found inside the cells in the acidic environment (pH 5.0) compared with those in the neutral one (pH 7.4). Based on all results, **I**_**2**_**-IR783-Mpip** could be a good candidate for tumour environment targeting agent for photodynamic treatment.

## Methods

### Synthesis of I_2_-IR783-Mpip

I_2_-IR783 (46.60 mg, 0.046 mmol) was dissolved in anhydrous DMF (4.0 mL), and then *N*-methylpiperazine (4.60 mg, 0.046 mmol) was added to the solution. Thereafter, the mixture was stirred at room temperature for 3 h under nitrogen atmosphere. After that, the solvent was removed under reduced pressure and the crude product was purified by column chromatography using CH_2_Cl_2_:MeOH (gradient from ratio 9:1 to 7:3) as the eluent to afford a blue solid (40.2 mg, 80.4%). ^1^H NMR (500 MHz, DMSO-d6): δ 7.93 (s, 1 H, Ar-CH), 7.70 (dd, *J* = *8*.*5*, 1.5, 1 H, Ar-CH), 7.60 (d, *J* = *12*.*0*, 1 H, CH), 7.15 (d, *J* = *8*.*5*, 1 H, Ar-CH), 5.98 (d, *J* = *13*.*0*, 1 H, CH), 4.04 (s, 2 H, N-CH_2_), 3.84 (s, 2 H, N-CH_2_), 2.69 (s, 2 H, N-CH_2_), 2.55 (s, 3 H, N-CH_3_), 2.40 (s, 2 H, N-CH_2_), 1.67–1.83 (m, 16 H). ^13^C NMR (125 MHz, DMSO-d6): δ 171.4, 166.8, 148.3, 143.5, 142.7, 142.0, 140.5, 136.8, 130.8, 127.1, 124.1, 113.9, 95.8, 89.6, 60.6, 50.9, 49.0, 47.5, 45.8, 42.7, 31.9, 28.32, 28.2, 25.5, 22.6. HRMS-ESI (m/z): the calculated value (calcd) for C_43_H_55_I_2_N_4_Na_2_O_6_S_2_^+^ [M^+^]: 1087.1442, found 1087.1449.

### General details for Vis-NIR and fluorescence measurements

Preparation of the stock solutions: The stock solution of **I**_**2**_**-IR783-Mpip** was prepared by dissolving 2.2 mg of **I**_**2**_**-IR783-Mpip** with methanol in a 3 mL standard micro volumetric flask (6.7 × 10^−4^ M). The UV-Vis-NIR and fluorescence measurements were performed by taking appropriate amount of this stock solution.

Vis-NIR absorption measurement: The stock solution of **I**_**2**_**-IR783-Mpip** (20 µL) was added to 3 mL of buffer solutions at various pH (pH 3-12) in a 3.5 mL quartz cuvette (final concentration = 4.5 µM). The UV-Vis-NIR absorption spectra were recorded by a Cary Series UV-Vis-NIR spectrophotometer (Agilent Tech, Santa Clara, CA, USA).

Fluorescence measurement: The stock solution of **I**_**2**_**-IR783-Mpip** (20 µL) was added to 3 mL of buffer solutions at various pH (pH 3–12) in a 3.5 mL quartz cuvette (final concentration = 4.5 µM). The fluorescence spectra were recorded by PTI QuantaMaster 500 – Near Infra-Red Photoluminescence System (HORIBA Scientific), using the following parameters: excitation wavelengths = 850 nm, excitation slit widths = 10 nm, and emission slit widths = 10 nm.

### Singlet oxygen generation

Singlet oxygen sensor green (SOSG) 10 μL (0.5 mM) was added into solutions of **I**_**2**_**-IR783-Mpip** (30 μM) in buffers pH 5.0, 7.0 and 9.0. Then, the solution was exposed to a NIR lamp (850 nm) at light intensity of 30 mW cm^−2^ for 5, 10, 20, 40, and 60 min. Buffers pH 5.0, 7.0 and 9.0 without **I**_**2**_**-IR783-Mpip** were also carried out as controls using the same method. Finally, fluorescent intensities of SOSG were measured at an excitation of 494 nm using Fluorescence microplate reader (Thermoscientific/VARIOSKAN LUX). Data were reported within error bars of three replicates experiments.

### Singlet oxygen quantum yield determination

Singlet oxygen generation of **I**_**2**_**-IR783-Mpip** was determined using 850 nm LED lamp. Sample solutions were prepared in phosphate buffer pH 5.0 and 7.4 at a concentration of 15 μM in a 6- well plate. 1,3-Diphenylisobenzofuran (DPBF, 1 mM) was prepared in DMSO and diluted to 70 μM in buffer solutions. The decrease in absorbance was measured at 418 nm every 1 min for 10 min by a BMG Labtech/SpeeTrostar Nano microplate reader. The rate of change of absorbance is plotted against irradiation time.

$${\Phi }_{{\rm{x}}}={\Phi }_{{\rm{st}}}({{\rm{grad}}}_{{\rm{x}}}/{{\rm{grad}}}_{{\rm{st}}})({{\rm{F}}}_{{\rm{st}}}/{{\rm{F}}}_{{\rm{x}}})$$

Φ_st_ represents the quantum yield of the standard; Φ_x_ represents the quantum yield of the unknown, and grad is the slope of the best linear fit. F stands for the absorption correction factor (F = 1−10^−abs^; abs represents absorbance), and subscripts x and st denote the unknown and the standard, respectively.

### Stability test

Solutions of **I**_**2**_**-IR783-Mpip** (15 µM) in 5% FBS in DMEM cell culture media at pH 5.0 and pH 7.4 were incubated at 37 °C for up to 168 h or 7 days. Vis-NIR spectra of the solutions were measured at different time points including 0, 1, 3, 6, 12, 24, 48, 72, 96, 120, 144, and 168 h respectively.

### Cell culture

Human hepatoma cancer cells (HepG2) and human embryonic kidney 293 (HEK-293) cells were cultured on 75 cm² culture flasks in Dulbecco’s Modified Eagle Medium/High glucose (DMEM/HIGH GLUCOSE, GE healthcare Life Sciences HyClone Laboratories) supplemented with 10% fetal bovine serum (FBS, Gibco) and 1% Penicillin Streptomycin Solution 100X (CORNING). All cells were cultured in a humidified incubator at 37 °C with 5% CO_2_.

### Cell survival assay of HepG2 cells in media 5.0

For cell survival assay, HepG2 cells were seeded into 96-well cell culture plates at 7 × 10³/well and incubated for 24 h at 37 °C under 5% CO_2_. After that, cell culture media were replaced with 5% FBS DMEM pH 5.0 at various incubation time (1, 3, 6, 12, 24, 36 h) and cells cultured in 5% FBS DMEM pH 7.4 were used as control. After incubation, the cell viabilities were measured using previously reported MTT protocol^26^. Briefly, the cells were washed with PBS (3 times) before being treated with methylthiazolyldiphenyl-tetrazolium bromide (20 µL, 0.5 mg/mL, Sigma-Aldrich) for 2 h. Then, culture media were replaced with DMSO and the cell viabilities were determined through UV-vis absorption of the resulting formazan at wavelength 560 nm using microplate reader (BMG Labtech/SPECTROstar Nano).

### Live cell imaging

HepG2 cells were seeded at a density of 7 × 10³ cells per well in 8-well Chambered Coverglass with non-removable wells (Nunc Lab-Tek II Chamber Slide) and incubated for 24 h at 37 °C under 5% CO_2_. After that, cell culture media were removed and solutions of **I**_**2**_**-IR783-Mpip** in DMEM with 5% FBS at pH 5.0 and pH 7.4 with final concentrations of 30 µM were added. After 1, 3, 6, 12 and 24 h incubation, the cells were washed three times with PBS to remove the non-uptake dye. Thereafter, the cells were stained with 1.0 µM Hoechst 33342 (Thermo Fisher Scientific) for 10 min. All cells were brought to image under Laser Scanning Confocal Microscope (LSCM, Nikon A1Rsi). **I**_**2**_**-IR783-Mpip** was excited by 641 nm laser and Hoechst 33342 was excited by 405 nm laser. A 60 X oil immersion objective lens was used.

### Colocalization study

After seeding the cells in an 8-well Chambered Coverglass and incubated for 24 h at 37 °C under 5% CO_2_, the cells were incubated with 30 μM of **I**_**2**_**-IR783-Mpip** in DMEM with 5% FBS at pH 5.0 and pH 7.4 for another 12 h, washed three times with PBS, and incubated with Lysotracker Green DND 26, Mitotracker Green FM (Thermo Fisher Scientific) for 20 min. The cells were washed again before staining with Hoechst 33342 for 10 min. After that, cells were visualized under LSCM: **I**_**2**_**-IR783-Mpip** was excited by 641 nm laser; Lysotracker and Mitotracker was excited by 488 nm laser; and Hoechst 33342 was excited by 405 nm laser. A 60 X oil immersion objective lens was used. To quantify the colocalization of **I**_**2**_**-IR783-Mpip** and organelles trackers, we obtained the intensities of the red and green channels from each pixel of the confocal image, corresponding to the excitations from **I**_**2**_**-IR783-Mpip** and organelles trackers, respectively, and calculate the Pearson’s correlation coefficient between the two intensities. The computation was carried out in python, using a built-in function from scipy statistical package.

### Flow cytometry

HepG2 and Hek293 cells were seeded into 6-well cell culture plates density of 2 × 10^5^ cells/well and incubated for 48 h at 37 °C under 5% CO_2_. After that, cell culture media were removed and solutions of **I**_**2**_**-IR783-Mpip** in DMEM with 5% FBS at pH 5.0 and pH 7.4 with final concentrations of 15 µM were added. After 12 h incubation, culture media were removed, and the cells were thoroughly washed with PBS. Thereafter, the cells were harvested by trypsinization whose action was stopped with 10% FBS in DMEM cell-culture media. The cells were then transferred into Eppendorf tubes and washed three times with ice cold PBS to remove the non-uptake dye by centrifugation at 800 g, 4 °C for 5 min and resuspended in 1 mL of fresh ice-cold PBS. Then 20,000 events (cells) were analyzed by flow cytometry using an Attune NxT Flow Cytometer (Life Technologies) using red excitation laser 637 nm and emission filter 780/60 nm.

### Light induced cytotoxicity

*In vitro* cell viability was measured using a standard methyl thiazolyltetrazolium (MTT) assay. HepG2 and Hek293 cells were seeded into 96-well cell culture plates at 7 × 10³ cells/well and incubated for 24 h at 37 °C under 5% CO_2_. After that, cell culture media were removed and solutions of **I**_**2**_**-IR783-Mpip** in DMEM with 5% FBS at pH 5.0 and pH 7.4 with various concentrations (0, 10, 20, 40, 50 µM) were added and the cells were continued incubation for another 6 h. Thereafter, the cells were irradiated by an 850 nm lamp with light intensity of 30 mW/cm^2^ for 30 min before re-incubation for another 12 h. In the case of deep-tissue simulation, a piece of 5-mm pork slice (sterile with 95% alcohol) was placed on the top of 96-well plate to insert between the cells and the lamp before irradiation. After incubation, the cell viabilities were measured using standard MTT protocol^26^ and the absorption of formazan was measured at wavelength 560 nm using microplate reader (BMG Labtech/SPECTROstar Nano).

For LIVE/DEAD viability/cytotoxicity assay, HepG2 cells were seeded into 6-well cell culture plates at a density of 2 × 10^5^ cells/well and incubated for 24 h at 37 °C under 5% CO_2_. After that, cell culture media were removed and solutions of **I**_**2**_**-IR783-Mpip** in DMEM with 5% FBS at pH 5.0 and pH 7.4 with final concentrations of 20 µM, 50 µM were added. After 6 h incubation, the cells were irradiated by an 850 nm lamp with light intensity of 30 mW/cm^2^ for 30 min before re-incubation for another 12 h. Thereafter, the cells were stained with 4 µM calcein AM and propidium iodide (PI) (Thermo Fisher Scientific) for 5 min, and then imaged by Fluorescence microscope (BioRad/Zoe) using 490 nm excitation and 515 nm emission filters for calcein AM and 535 nm excitation and 615 nm emission filters for PI.

### Cellular reactive oxygen production

HepG2 cells were seeded at a density of 7 × 10³ cells per well in 8-well Chambered Coverglass with non-removable wells and incubated for 24 h at 37 °C under 5% CO_2_. After that, cell culture media were removed and solutions of **I**_**2**_**-IR783-Mpip** (20 μM) in DMEM with 5% FBS at pH 5.0 and pH 7.4 were added. After 6 h incubation, the cells were washed three times with PBS to remove the non-uptake dye and then 20 µM of 2,7-dichloro-dihydro-fluorescein diacetate (DCFH-DA, Sigma-Aldrich) was added into the cells. After incubation for 1 h, the cells were washed three times with PBS to remove the non-uptake DCFH-DA dye before irradiation with an 850 nm lamp for 20 min. Before imaging, the cells were stained with 1.0 µM Hoechst 33342 (Thermo Fisher Scientific) for 10 min then fluorescence of the resulting 2,7-dichloro-dihydro-fluorescein (DCF) was monitored using 488 nm excitation laser and Hoechst 33342 was excited at 405 nm under Laser Scanning Confocal Microscope (Nikon A1Rsi). A 60 X oil immersion objective lens was used.

For quantitative analysis, HepG2 cells were seeded into 96-well cell culture plates at 7 × 10³/well and incubated for 24 h at 37 °C under 5% CO_2_. After that, cell culture media were removed and solutions of **I**_**2**_**-IR783-Mpip** (20 μM) in DMEM with 5% FBS at pH 5.0 and pH 7.4 with various concentration (0, 10, 20, 40, 50 µM) were added. After 6 h incubation, the cells were washed three times with PBS before being irradiated by 850 nm lamp for 20 min. Thereafter, 20 µM of DCFH-DA was added into each well and the cells were continue incubated for another 45 min. Fluorescent end point of the DCF production was recorded at 495 nm excitation and 529 nm emission using a Fluorescence microplate reader (Thermo Fisher Scientific/VARIOSKAN LUX).

### Statistical analysis

Data are illustrative of three independent experiments and presented as the mean of four individual observations (n = 4) with the standard deviation (mean ± SD). The statistical analysis was performed using one-way ANOVA followed by Tukey’s post-hoc analysis. P values < 0.05 were considered to indicate significance (*P < 0.05, **P < 0.01, ***P < 0.001).

## Supplementary information

Supplementary informations.
