# Supplementary material for: Near-Infrared Fluorescent pH Responsive Probe for Targeted Photodynamic Cancer Therapy
Source: Sci Rep. 2020 Jan 28;10:1283. doi: 10.1038/s41598-020-58239-5 (PMC6987190; doi:10.1038/s41598-020-58239-5)
Supplement: Supplementary file 1 — Supplementary informations. [file 41598_2020_58239_MOESM1_ESM.pdf]

Near-Infrared Fluorescent pH Responsive Probe for Targeted Photodynamic Cancer Therapy

Supporting Information

Siriwalee Siriwibool,[a,†] Nantawat Kaekratoke,[a,†] Kantapat Chansaenpak,[b] Kittipan Siwawannapong,[a] Pannipa Panajapo,[a] Kritsana Sagarik,[a] Parinya Noisa,[c] Rung-Yi Lai [a] and Anyanee Kamkaew[*,a]

[a] School of Chemistry, Institute of Science, Suranaree University of Technology, Nakhon Ratchasima, Thailand 30000

[b] National Nanotechnology Center, National Science and Technology Development Agency, Thailand Science Park, Pathum Thani, Thailand 12120

[c] Laboratory of Cell-Based Assays and Innovations, School of Biotechnology, Institute of Agricultural Technology, Suranaree University of Technology, Nakhon Ratchasima, Thailand 30000

1. ^{1}H NMR, ^{13}C NMR, and HRMS results of **I$_2$-IR783-Mpip** ……………………………… …..2

2. Total energy from DFT calculation of **I$_2$-IR783-Mpip**………… …………………….............3

3. Singlet oxygen ($^1O_2$) generation assay of **I$_2$-IR783-Mpip**……………………………………4

4. Relative cell viability in media pH 5.0…………………………..……………………………4

5. Organelle colocalization study………………………………………………………………….5

6. Time dependent cellular uptake…………………………………………………………………6

7. Relative cell viabilities of HEK-293 cells after incubation with **I$_2$-IR783-Mpip**………………6

8. Light Induced cytotoxicity of HepG2 cells after incubation with **I$_2$-IR783-Mpip**……………..7

9. Cellular reactive oxygen production of **I$_2$-IR783-Mpip**……………………………………......7

(a)

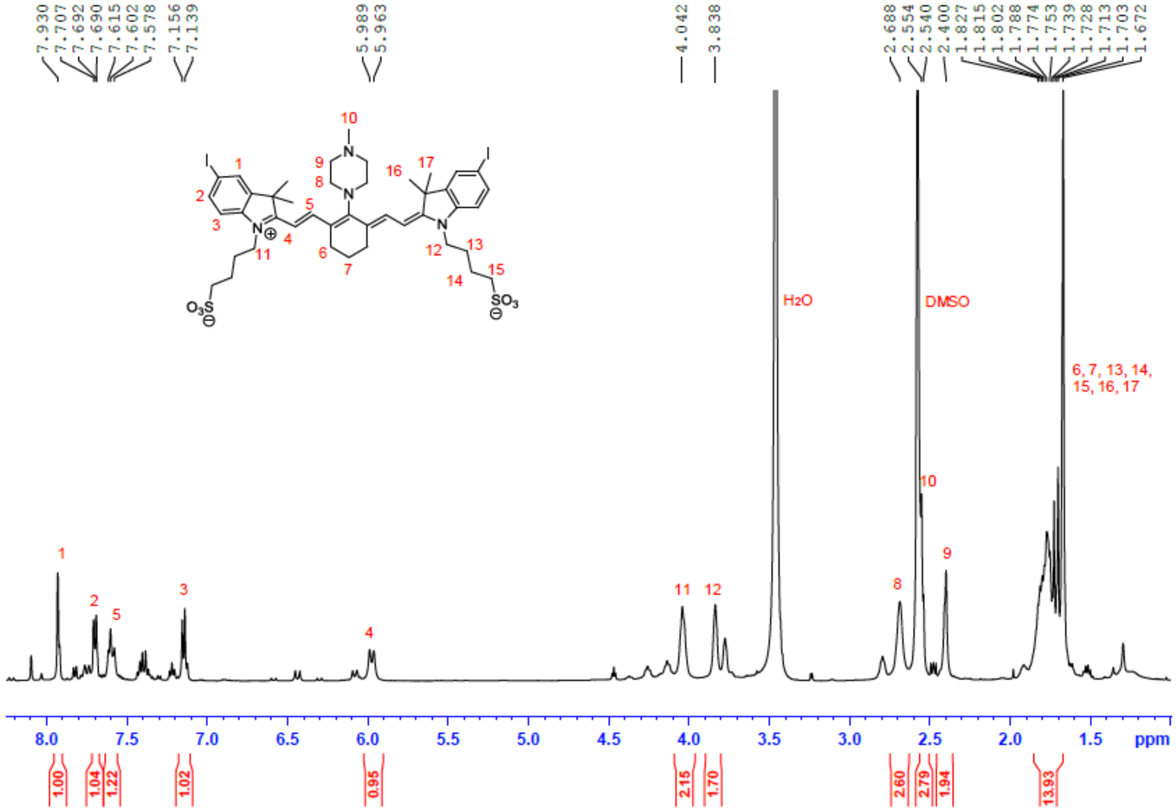
7.930
7.707
7.692
7.690
7.615
7.602
7.578
7.156
7.139
5.989
5.963
4.042
3.838
2.688
2.554
2.540
2.400
1.827
1.815
1.802
1.788
1.774
1.753
1.739
1.728
1.713
1.703
1.672
H2O
DMSO
6, 7, 13, 14, 15, 16, 17
ppm
8.0
7.5
7.0
6.5
6.0
5.5
5.0
4.5
4.0
3.5
3.0
2.5
2.0
1.5
1.00
1.04
1.22
1.02
0.95
2.15
1.70
2.60
2.79
1.94
13.93

(b)

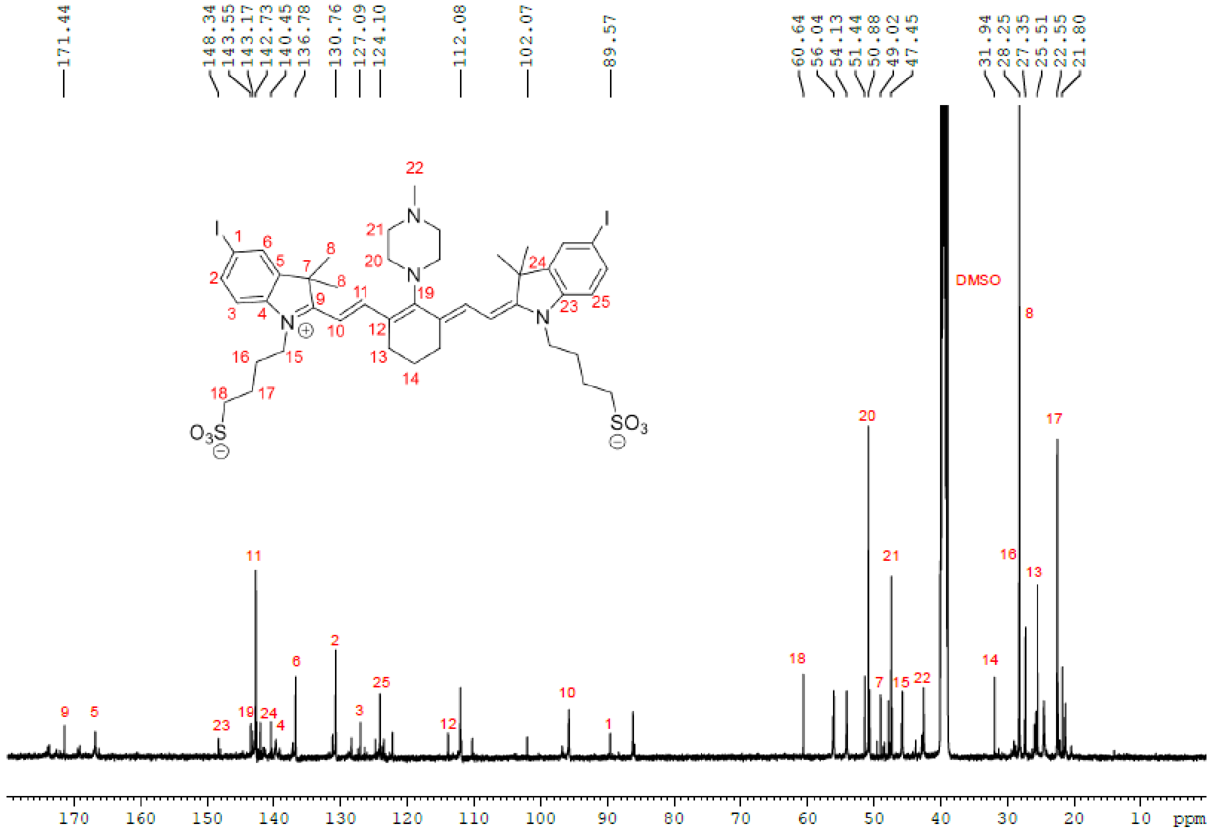
171.44
148.34
143.55
143.17
142.73
140.45
136.78
130.76
127.09
124.10
112.08
102.07
89.57
60.64
56.04
54.13
51.44
50.88
49.02
47.45
31.94
28.25
27.35
25.51
22.55
21.80
DMSO
ppm
170
160
150
140
130
120
110
100
90
80
70
60
50
40
30
20
10

(c)

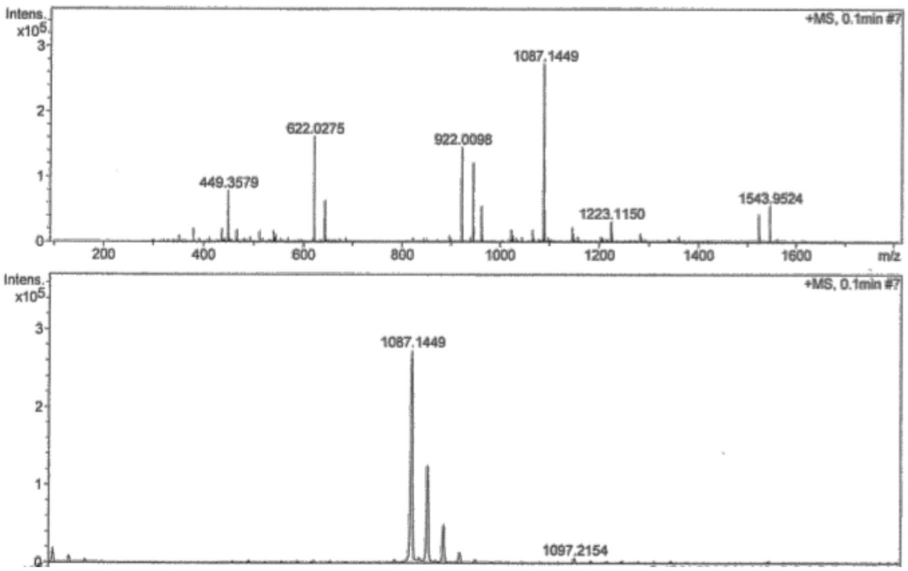

Figure S1. (a) ^{1}H-NMR spectrum of **I$_2$-IR783-Mpip**. (b) ^{13}C-NMR spectrum of **I$_2$-IR783-Mpip**. (c) HRMS spectrum of **I$_2$-IR783-Mpip**.

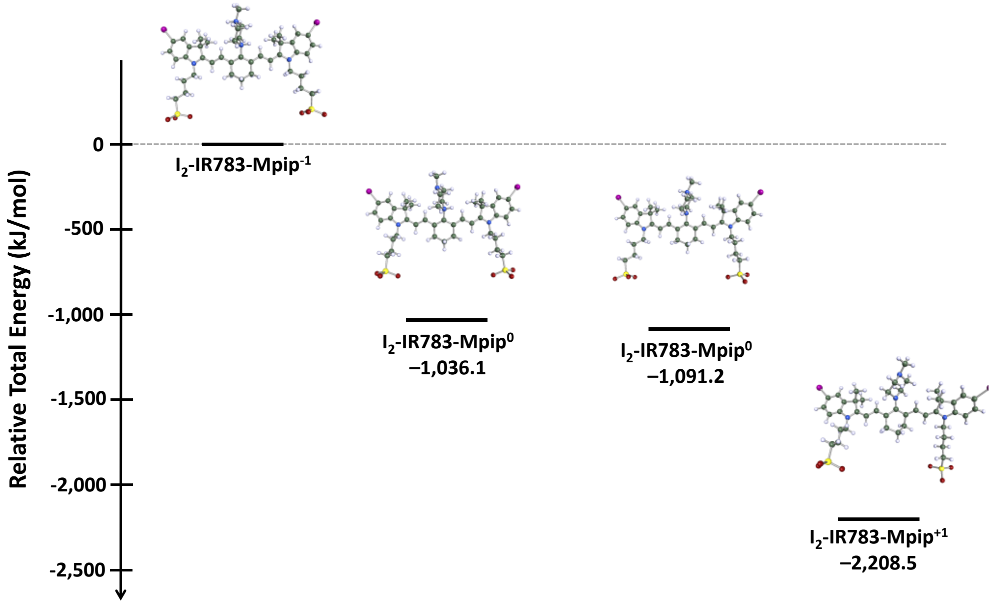

Figure S2. Relative total energy of possible structures of I$_2$-IR783-Mpip^0 (2 forms) and I$_2$-IR783-Mpip^{+1} compared to I$_2$-IR783-Mpip^{-1} obtained from DFT/6-311G geometry optimizations in the COSMO phase, dielectric environment 78.

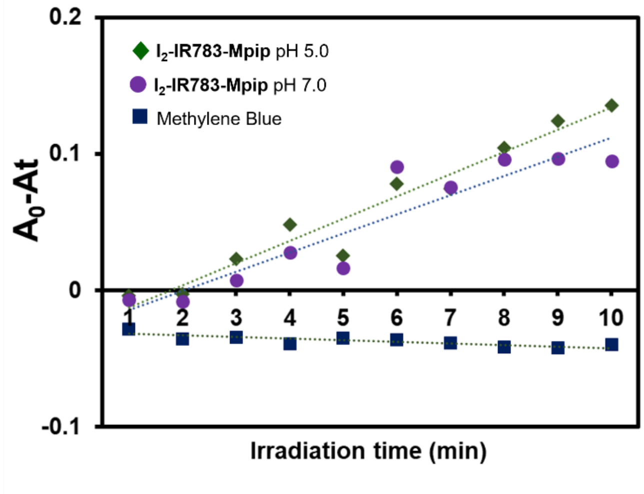

Figure S3. Singlet oxygen ($^1O_2$) generation assay as deduced by of change in absorbance of DPBF (1,3-diphenylisobenzofuran; added *in situ*) at 418 nm.

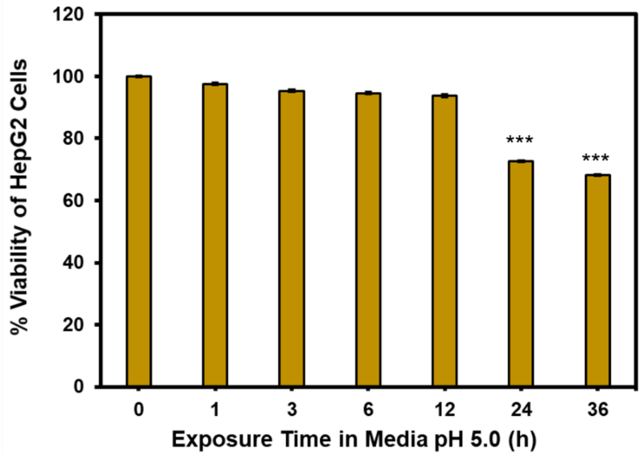

Figure S4. Relative cell viability of HepG2 cells after exposure to DMEM media pH 5.0 supplemented with 5 % FBS for 0-36 h. Statistical analysis are based on Student's t-test (*P < 0.05, **P < 0.01, ***P < 0.001).

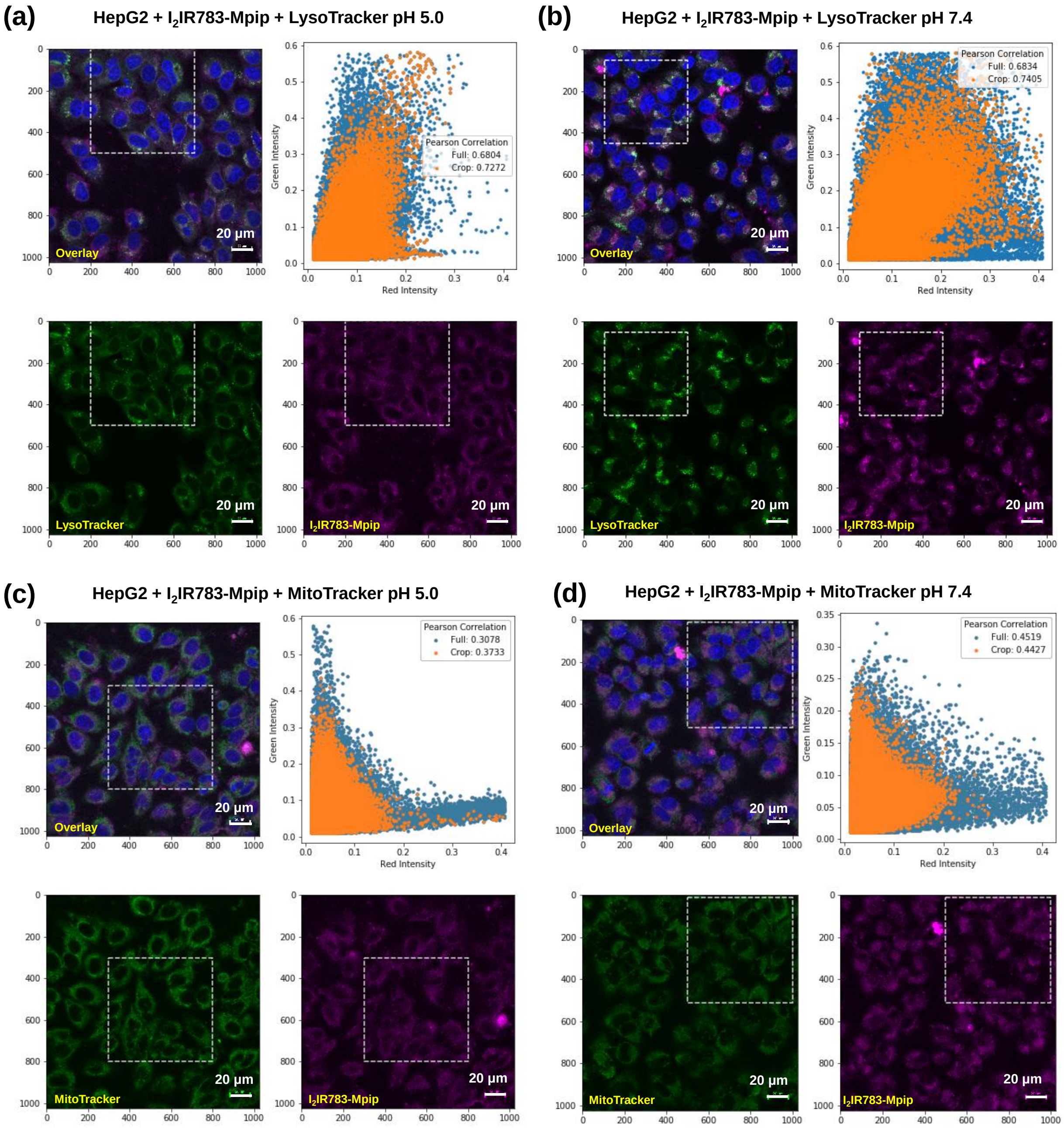

Figure S5. Organelle colocalization study in **I2IR-783-Mpip** with LysoTracker in media pH 5.0 (a) and pH 7.4 (b) and MitoTracker Green in media pH 5.0 (c) and pH 7.4 (d).

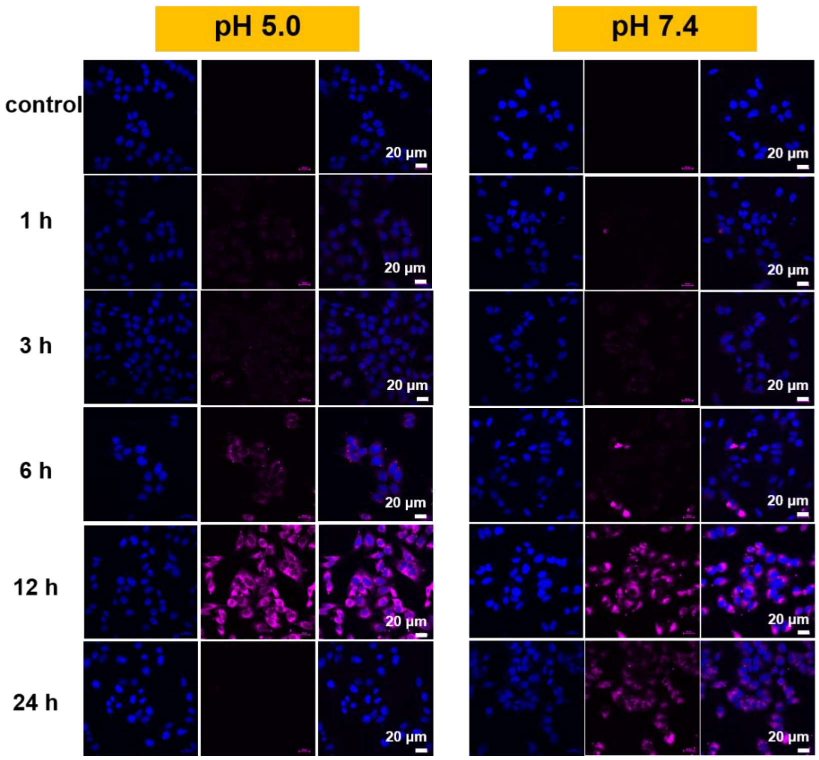

Figure S6. Time dependent cellular uptake in both media pH 5.0 and pH 7.4. Confocal images of HepG2 cells at selected time point (1, 3, 6, 12, 24 h) post incubation of **I_2-IR783-Mpip** (30 μM). **I_2-IR783-Mpip** fluorescent signal was displayed in purple, DAPI nucleus co-staining signal was showed in blue. Scale bars = 20 μm.

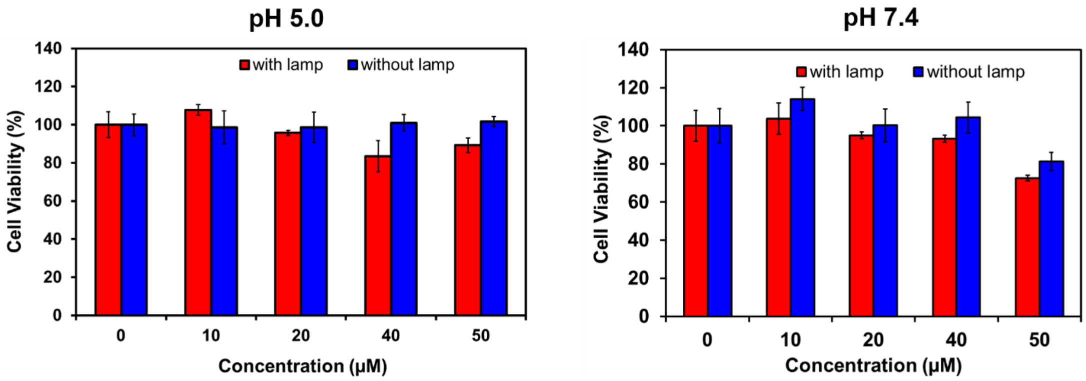

Figure S7. Relative cell viabilities of HEK-293 cells after incubation with **I_2-IR783-Mpip** at different concentrations (0 – 50 μM) at pH 5.0 and pH 7.4 for 6 h and with and without being exposed to 850 nm lamp (light intensity 30 mW/cm^2) for 30 min.

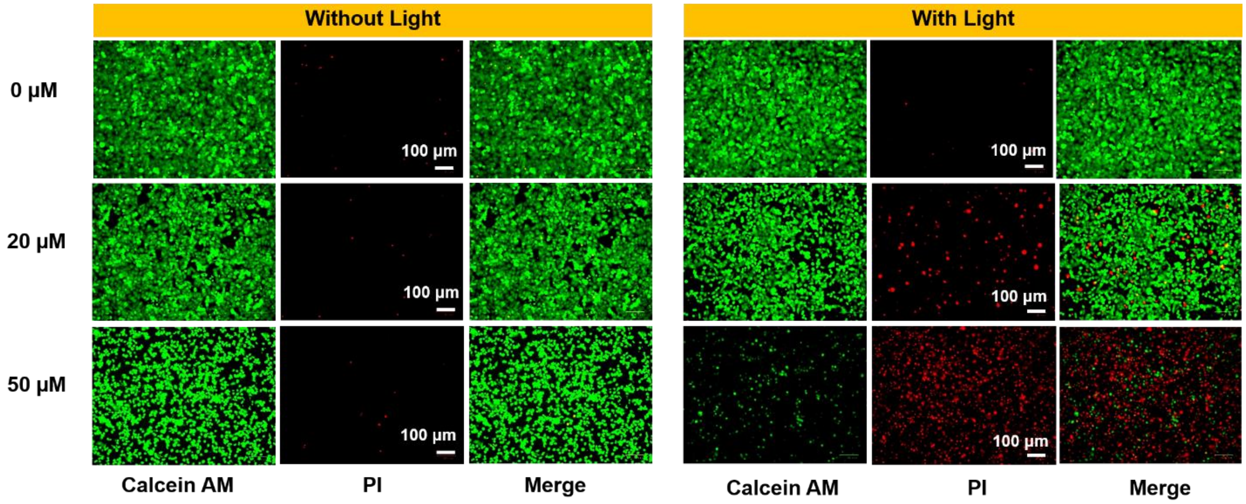

Figure S8. Fluorescence images of HepG2 cells after incubation with **I_2-IR783-Mpip** in DMEM pH 7.4 at concentrations of 20 and 50 μM for 6 h with and without light exposure (850 nm). Live and dead cells were stained with Calcein AM and PI as presented in green and red colors in the images, respectively. Scale bars = 100 μm.

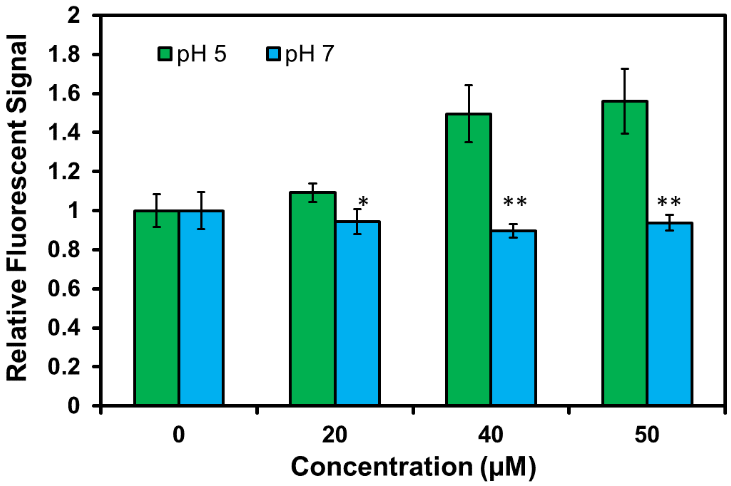

Figure S9. Relative fluorescence signal of DCF in HepG2 cells after incubation with **I_2-IR783-Mpip** at different concentrations (0 – 50 μM) at pH 5.0 and pH 7.4 for 6 h and with and without being exposed to 850 nm lamp (light intensity 30 mW/cm^2) for 30 min. Statistical analysis was performed using the Student's two tailed t-test (*$P < 0.05$, **$P < 0.01$, ***$P < 0.001$).
